# Targeting N17 domain as a potential therapeutic target for the treatment of Huntington disease: An opinion

**DOI:** 10.17179/excli2021-3670

**Published:** 2021-06-09

**Authors:** Vishal Kumar, Arti Singh

**Affiliations:** 1 Department of Pharmacology, ISF College of Pharmacy, Moga-142001, Punjab, India; 2 Affiliated to IK Gujral Punjab Technical University, Jalandhar, Punjab-144603, India

## ⁯

***Dear Editor, ***

Huntington's disease (HD) is an autosomal dominant, inherited neurodegenerative disease. Clinical symptoms may include such as chorea, depression, and cognitive decline, begin at age of 40 years and the disease becomes severe up to the age of approximately 65 years (Ignácio et al., 2019[8]). The estimated prevalence of HD is 10 in 100000 people worldwide. HD is mainly characterized by cognitive decline followed by motor and psychiatric dysfunctions. Initially, HD patients develop progressive movements of the extremities (Veldman et al., 2015[19]) similarly; they also develop akinesia (loss of mobility), bradykinesia in the later stage (slowness of movement), rigidity, and gait abnormality. The pathological hallmark of HD is an expansion of CAG (cytosine, adenine, and guanine) triplet on exon-1 of huntingtin protein (Htt) (Richards et al., 1981[15]).

Normal Htt has 35 CAG repeats, whereas mutant htt having >40 sCAG repeats, patients with 36-39 CAG repeat expansion are at risk of HD (Reuter et al., 2008[14]). The first definitive depiction of HD was given by Dr. George Huntington in his article entitled "On Chorea" in 1872. Dr. Huntington identifies the basic features of the disease; such as inherited nature, symptoms in adult life, and progressive nature of the disease (Bombard, 2008[3]). Medium Spiny neurons (MSNs) (positioned in the striatum) and cortical pyramidal neurons (located in the cerebral cortex, hippocampus, and amygdala) that are involved in the motor functions are most susceptible to degeneration in HD (Vonsattel et al., 2011[20]).

At present, there is no effective treatment available for the management of HD. Currently, only symptomatic therapeutic strategies are available for the treatment of HD, which focuses on neurological and psychiatric symptoms that aim to improve the quality of life (Hersch and Rosas, 2008[7]). Tetrabenazine (TBZ) is the only drug approved by US-FDA in 2008 for the treatment of HD. It acts via the blocking of vesicular monoamine transporter-2 in humans resulting reduction in monoamines including dopamine (Kaur et al., 2016[9]).

The N17 domain in Htt is a multifunctional localization signal which forms an amphipathic alpha-helix and is involved in post-translational modifications such as acetylation (Thompson et al., 2009[18]), phosphorylation (Atwal et al., 2011[1]), and sumoylation (Steffan et al., 2004[17]). Most notably N17 domain is highly expressed in the striatum region of the brain. Evidence suggests that N17 domain mediates localization of endoplasmic reticulum, mitochondria and Golgi apparatus (Orr et al., 2008[12]; Rockabrand et al., 2007[16]), as a cytoplasmic retention domain (Yan et al., 2011[21]), as a translocated promoter region (TPR)-dependent nuclear export signal (Cornett et al., 2005[5]) and as a membrane-binding domain that localizes endoplasmic reticulum(Atwal et al., 2007[2]; Maiuri et al., 2013[10]). It is a principal regulator of stabilization and localization of Htt which contributes to the pathogenic mechanism caused by the mutant Huntingtin (mHtt) (Steffan et al., 2004[17]). It plays a key role in both the cytoplasmic membrane association domain and nuclear export signaling. Study revealed that phosphorylation of serine 13 and serine 16 within the N17 domain may reduce aggregation and toxicity of Htt in both *in vitro* (Thompson et al., 2009[18]) as well as *in vivo* (Gu et al., 2009[6]). Huntingtin protein localization may alter by the ROS (Reactive oxygen species). 

**Figure 1:**
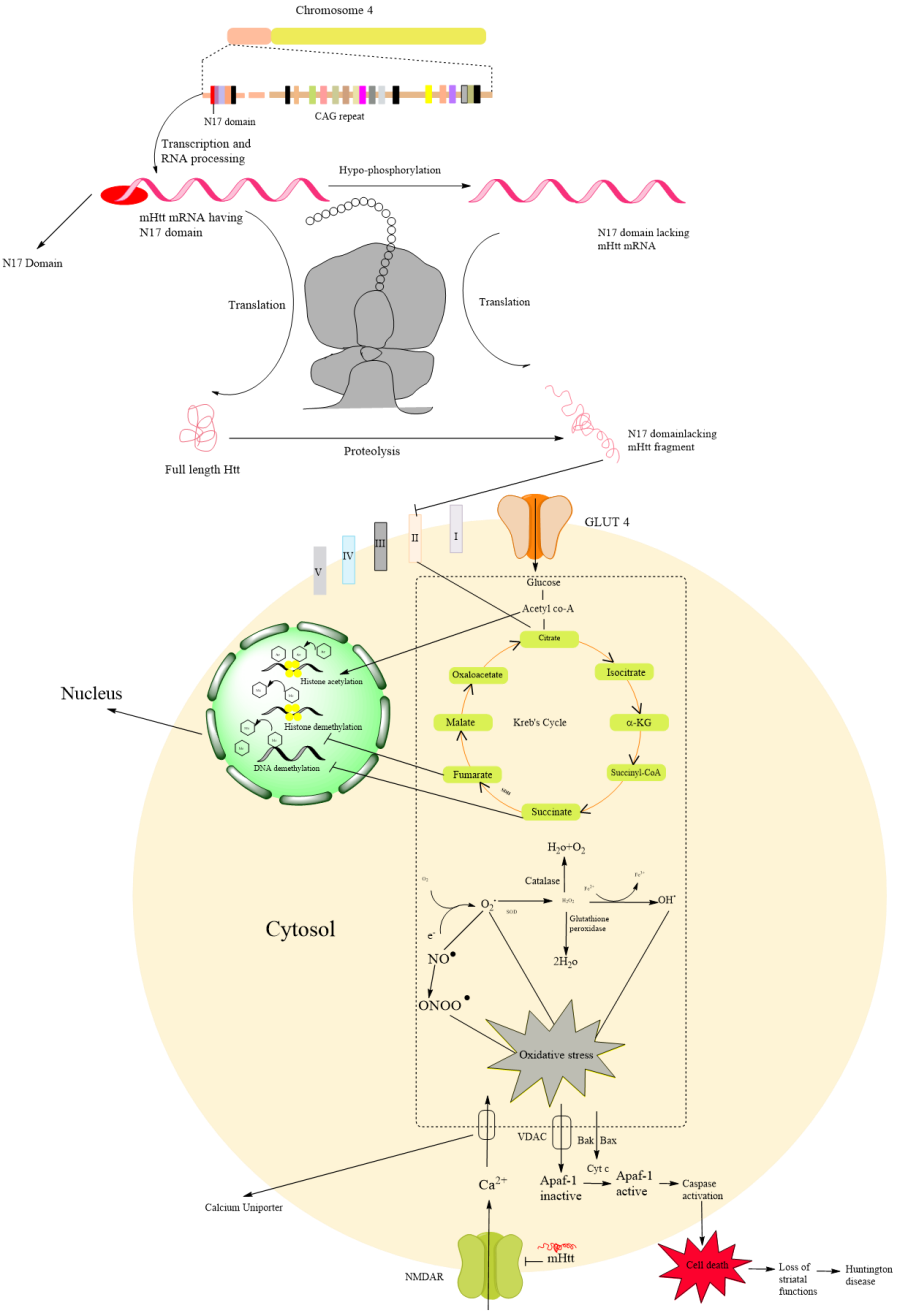
Schematic representation Huntingtin structure and N17 domain lacking mutant Huntingtin mediated Huntington pathogenesis through mitochondrial dysfunction Huntingtin (Htt) gene located on the chromosome 4, primarily it generates initial RNA having N17 domain which is further converted in mutant Htt mRNA lacking N17 domain by the hypo-phosphorylation. Further, it undergoes the translation process and formed the N17 domain lacking Huntingtin protein. It undergoes proteolytic cleavage results in the formation of mutant huntingtin fragments which cause mitochondrial dysfunction via the generation of reactive oxygen species (ROS). Primarily Htt affects mitochondrial complex I, II, III, and IV which are involved in the respiratory chain results in decreased ATP production via causing dysfunction in succinate dehydrogenase (SDH). Huntingtin fragments accumulate in the nucleus and directly affect histone acetylation and trigger histone and DNA methylation. Succinate and fumarate are the intermediated product of Kreb's cycle which is primarily involved in the ATP production, it inhibits the histone and DNA methylation in the nucleus. Reactive oxygen species are associated with oxidative stress. The calcium uniporter increases the intracellular concentration of calcium. Due to oxidative stress, the VDAC transporter transports the inactive Apaf-1 which is further activated by the cytochrome C resulting in caspase activation that causes cell death of medium spiny neurons.

Based on the above discussion we hypothesized that N17 domain expression in Htt, may reduce the accumulation and increase the nuclear clearance of mHtt. Mutant Huntingtin accumulated in both the nucleus and cytoplasm. A study in invertebrates (zebrafish) revealed that zebrafish without the N17 domain showed an early onset of symptoms, whereas zebrafish with the N17 domain showed a delayed onset of symptoms. Therefore, the authors hypothesize that the expression of N17 domain in mHtt reduces the accumulation of mHtt and its toxicity in both nucleus and cytoplasm (Veldman et al., 2015[19]). Mitochondrial accumulation of mutant huntingtin may alter the electron transport chain while nuclear accumulation of mutant huntingtin alters the gene expression and translation of Htt (Zheng et al., 2018[22]). Another study illustrates that phosphorylation of the N17 domain prevents nuclear export of huntingtin during transient stress response events as well as releases Huntington from the endoplasmic reticulum (ER) to allow nuclear entry (Maiuri et al., 2013[10]). Mutant Htt primarily affects mitochondrial complex II and causes calcium abnormalities through interaction with the outer mitochondrial membrane (Choo et al., 2004[4]). Similarly, it decreases intracellular ATP and increases reactive oxygen species (ROS) production. The enzyme involved in the tricarboxylic acid (TCA) cycle i.e. aconitase is highly susceptible to superoxide-mediated inactivation of ATP production in mitochondria (Mochel and Haller, 2011[11]). Superoxide is further converted into hydrogen peroxide in the presence of superoxide dismutase (SOD) followed by the production of hydroxyl ions. Cytochrome C is released from the mitochondria upon increase production of ROS which further leads to caspase activation, results in apoptosis in striatal neurons (Redza-Dutordoir and Averill-Bates, 2016[13]) (Figure 1(Fig. 1)). 

Hence it is suggested that Htt protein having N17 domain in zebrafish, shows the reduction in disease progression. We have critically reviewed the literature and discussed the possible mechanism underlying the N17 domain lacking mHtt mediated mitochondrial dysfunction and neuronal cell death in HD. The N17 domain could be a potential therapeutic target for the development of the new therapeutic strategy for the treatment of Huntington disease.

## Acknowledgement

Authors are thankful to the Department of Pharmacology, ISF College of Pharmacy, Moga for providing support.

## Conflict of interest

The authors declare that there is no conflict of interest.
